# Leiomyoma Causing Disease in a Wild Adriatic Sturgeon ( *Acipenser naccarii* , Bonaparte, 1836)

**DOI:** 10.1111/jfd.14128

**Published:** 2025-04-05

**Authors:** A. Marsella, T. Pretto, C. Samassa, P. Tedesco, F. Pascoli, L. Congiu, A. Gustinelli, F. Borghesan, A. Toffan

**Affiliations:** ^1^ Istituto Zooprofilattico Sperimentale Delle Venezie Legnaro Italy; ^2^ Biology Department, CoNISMa Padua University Padua Italy; ^3^ Department of Veterinary Medical Sciences Alma Mater Studiorum University of Bologna Ozzano Emilia (BO) Italy; ^4^ Freelance Biologist and Aquaculture Consultant Italy

**Keywords:** *Acipenser naccarii*, Adriatic sturgeon, leiomyoma, *Leptorhynchoides*

## Abstract

The Adriatic sturgeon, *Acipenser naccarii* , is an endemic species to the Adriatic Sea basin classified as *critically endangered* according to the IUCN Red List. Limited information is available regarding the health status and diseases affecting this species, particularly in the wild. In September 2023, a post‐spawning female Adriatic sturgeon was found along the edges of the Adige River. The fish displayed abnormal swimming, cutaneous haemorrhages, extreme emaciation and died shortly after its finding. At necropsy, the stomach appeared enlarged with a firm texture and a severe restriction of the lumen due to the thickening of the gastric wall. A gastric leiomyoma was characterised by histology, and this represents the first description of such neoplasia in *A. naccarii* . In the intestine, a severe infestation with roundworm belonging to the phylum Acanthocephala was responsible for severe haemorrhagic enteritis. The parasites were identified as belonging to the species *Leptorhynchoides polycristatus* , providing the first description of such a parasite in Italy. Finally, genetic analyses were performed in order to assess genetic purity and relatedness with captive breeding stocks. The results showed that the individual did not cluster with any captive identified groups, supporting the likelihood of a pure and wild‐origin Adriatic sturgeon.

## 1 Introduction

The Adriatic sturgeon, *Acipenser naccarii* Bonaparte 1836, is an endemic species to the Adriatic Sea basin and is currently classified as *Critically Endangered* according to the International Union for Conservation of Nature (IUCN) Red List. Once abundant in Italian rivers, *A. naccarii* has suffered severe population declines due to habitat loss, river regulation, pollution and overfishing. This fish is nationally recognised for its ecological and evolutionary significance, beside its traditional importance in some regions linked to caviar production. Except for a few recent pieces of evidence of reproduction occurring in the wild (Congiu et al. 2021), the species survival mostly depends on restocking programmes conducted by releasing juveniles generated from adult breeders reared in aquaculture establishments. In fact, in the last years, several restocking efforts have been supported by EU projects (i.e., LIFE04 NAT/IT/000126; LIFE03 NAT/IT/000113) by releasing individually marked sturgeon in Italian waterbodies (Arlati and Poliakova 2008; Barca et al. 2022). In order to monitor the purity of breeders used in restocking programmes as well as to monitor possible inbreeding phenomena, the genetic pedigrees of all captive Adriatic sturgeon used in Italy are already known (Barca et al. 2022; Boscari and Congiu 2014). Furthermore, for the same reason, every *ex situ* population should be investigated genetically to determine if it is a representative sample of the available genetic diversity or can represent a possibility of enrichment of the genetic stock already known (Christie et al. 2012; Doukakis et al. 2010; Russello and Amato 2004; Theodorou and Couvet 2010). Besides already identified threats to the species such as poaching, damming, pollution and invasive species, very little information is available regarding the diseases that can affect this species. Bigarré et al. (2017) reported the detection of Acipenser Iridovirus‐European (AcIV‐E) in an Adriatic sturgeon sample collected in 2014 from farmed adults showing clinical signs. Similarly, Bondavalli et al. (2024) detected AcIV‐E in 2022 and 2023 in Adriatic sturgeon samples collected following mortality events in sturgeon farms located in north‐western Italy. These findings strongly suggest the susceptibility of Adriatic sturgeon to the AcIV‐E infection, which can then represent a threat to the species as already documented for other sturgeon species (in particular for the *Acipenser gueldenstaedtii* ). Conversely, some bacterial species have been reported to cause disease in farmed Adriatic sturgeon, in particular *Aeromonas hydrophila* (Santi et al. 2019) and *Streptococcus iniae* (Mugetti et al. 2022). Notably, the latter caused significant mortalities in the reported case, inducing between 6.4% and 11.2% monthly mortality. All these reports refer to farmed Adriatic sturgeon, because outbreaks in the wild are extremely difficult to be identified and investigated. For this reason, it is extremely valuable to investigate every single occurrence of wild dead sturgeon reported. This study therefore describes the clinical and anatomopathological findings as well as the laboratory results of a case of disease involving an adult specimen of Adriatic sturgeon retrieved in Italy in the wild.

## 2 Case Description

In September 2023, an adult Adriatic sturgeon measuring 140 cm standard length was found in distress in the north‐eastern Italy along the edges of the Adige River, nearby its estuary. No adverse weather conditions were reported during the previous 2 weeks as well as other relevant hydrological events or water anomalies. Water temperature was 26°C on average and salinity 25 ppt when the fish was observed. The presence of other aquatic species such as mullets and blue crab not affected by any apparent disease was reported in the same river. The fish displayed abnormal swimming (surfing and colliding with riverbanks), some cutaneous haemorrhages and extreme emaciation. Because of the ecological relevance of the species, the rescue of the subject by capturing it and introducing it into a commercial sturgeon aquaculture establishment was attempted. Unfortunately, the animal died a few hours after the transport and the carcass was frozen for 2 weeks before being consigned to the laboratory for the necropsy.

## 3 Anatomopathological Examination

The fish was thawed and subjected to necropsy according to standard procedure. The specimen proved to be a mature female, as confirmed also by wet mount examination of the gonad. Standard length (SL) was 140 cm, while total length was 150 cm. The fish weighted 13.5 kg and the *K* index was calculated according to the following formula by Fulton (1904):

K=Weightg*100Lcm^3

**FIGURE 1:**
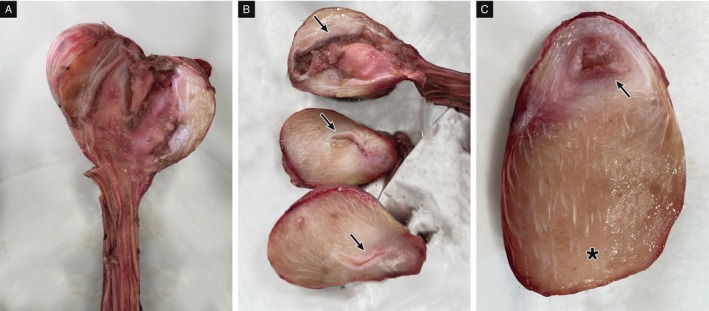
(A–C) Macroscopical examination of the stomach through sagittal slices. Severe reduction of gastric lumen (arrow) and thickening of the gastric muscular layer (asterisk) can be appreciated.

Although not standardised for the Adriatic sturgeon, the *K* value of 0.5 indicated a bad nutritional status, confirmed by the emaciation appreciated at gross examination. No tag was retrieved suggesting that the fish did not originate from restocking activities. Through external examination, it was possible to observe erosive lesions with loss of tissue in particular at the level of the caudal fin. As reported by anglers, several blue crabs were observed feeding on the sturgeon body, and these lesions were probably linked to their presence. No other external lesions were appreciated. Internally, it was possible to observe a slight colliquation and congestion of the liver coupled with a severe enlargement of the gallbladder consistent with starvation. The heart ventricular wall proved thinner than expected. The stomach appeared as a firm mass with semi‐elastic consistency and the size of a pomegranate. After a transversal cut, it was possible to observe a severe thickening of the wall and the presence of a mass of the same colour and texture as the wall tissue protruding in the stomach lumen. The almost complete occlusion of the gastric lumen was observed (Figure 1C).

**FIGURE 2:**
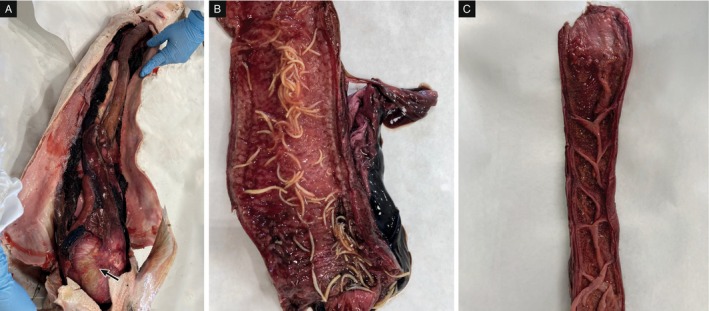
Macroscopical examination of the intestine. (A) Arrow highlights the location of the neoplasia. Severe congestion of the intestine can be observed. (B) Several roundworm specimens are present within the lumen of the anterior intestine. (C) No parasites are observed in the spiral valve.

The whole intestine appeared severely congested (Figure 2A). Once opened, the intestinal mucosa appeared haemorrhagic and severely thickened. In the anterior part of the intestine before the spiral valve, a massive infestation of round worms was detected with at least 104 individuals counted (Figure 2B,C).

No other internal lesions were detected. Based on gross findings, the disease was linked to the gastric occlusion and massive parasitological infestations. Due to the long frozen conservation and since no suspicion of infectious disease was raised, no sampling was performed for bacteriological analysis.

## 4 Virological Analysis

Although no infectious disease was suspected, sampling for virological analysis was attempted to collect data regarding viral infection prevalence in Adriatic sturgeon. At necropsy, gill samples were collected and tested for AcIV‐E detection by qPCR according to the protocol described by Bigarré et al. (2017) and by viral isolation on WSSK‐1 cell line for *Acipenser herpesvirus* according to the protocol described by Ciulli et al. (2020). Both analyses yielded negative results.

## 5 Parasitological Analysis

Intestinal parasites were examined through wet mount and were confirmed to belong to the phylum Acanthocephala. Some specimens were fixed in either 70% ethanol or 10% buffered formalin for further processing.

For observation in light microscopy, ethanol‐fixed parasites were clarified in Amman's lactophenol and measured using a digital Camera Nikon and the Imaging software NIS‐Elements–Nikon following the keys reported by Amin et al. (2013).

The measurements (in mm unless otherwise stated) are given as a range (min–max) followed by the mean and standard deviation. The width measurements refer to the widest region of the body (Table 1).

**TABLE 1:** Measurements of anatomical structures of the examined specimens of *Leptorhynchoides polycristatus.* Measures are expressed in micrometres unless otherwise stated.

	Male (*n* = 2)				Females (*n* = 4)		
	Length		Width		Length	Width	
	Min–max (mean ± SD)						
Trunk	11.43–13.45 mm (12.44 ± 1.43)		1352.11–1359.52 (1355.82 ± 5.24)		11.03–17.9 mm (14.15 ± 2.8)	1057.77–1740.94 (1413.96 ± 287.81)	
Proboscis	2480.63–2530.94 (2505.79 ± 35.57)		437.98–450.85 (444.42 ± 9.1)		2949.11–3216.02 (3113.32 ± 114.76)	365.41–487.49 (427.84 ± 67.38)	
Proboscis receptacle	2415.52–2626.51 (2521.02 ± 149.19)		n.a.	n.a	2548.79–3029.2 (2726.53 ± 263.44)	n.a.	
Apical hooks	70.83–83.13 (76.98 ± 8.7)		12.55–14.08 (13.32 ± 1.08)		75.49–105.67 (90.64 ± 14.93)	12.65–28.01 (20.65 ± 6.3)	
Central hooks	75.64–100.67 (88.16 ± 17.7)		15.77–16.76 (16.27 ± 0.7)		89.70–115.29 (102.03 ± 10.53)	13.55–32.19 (22.23 ± 7.71)	
Basal hooks	54.57–60.51 (57.54 ± 4.2)		9.15–10.2 (9.68 ± 0.74)		72.15–87.04 (80.52 ± 7.21)	14.04–27.57 (18.31 ± 6.24)	
Neck	281.39–350.2 (315.8 ± 48.66)		283.8–297.09 (290.45 ± 9.4)		303.31–443.26 (363.44 ± 59.23)	323.86–382.35 (347.05 ± 27.97)	
Longer lemniscus	4913.83–4939.97 (4926.9 ± 18.48)		155.99–292.49 (224.24 ± 96.52)		1807.55	118.51	n.a.
Shorter lemniscus	2266.1–2454.69 (2360.4 ± 133.35)		151.92–225.85 (188.89 ± 52.28)		1116.59–2570.45 (2068.31 ± 824.63)	139.28–392.55 (238.81 ± 135.06)	
Anterior testis	767.03	n.a.	611.74	n.a.	—	—	
Posterior testis	667.06	n.a.	444.08	n.a.	—	—	
Eggs	—	—	—	—	138.80–157.85 (147.69 ± 6.11)	25.1–29.02 (27.12 ± 1.31)	

*Note:* n.a.: Not available.

**FIGURE 3:**
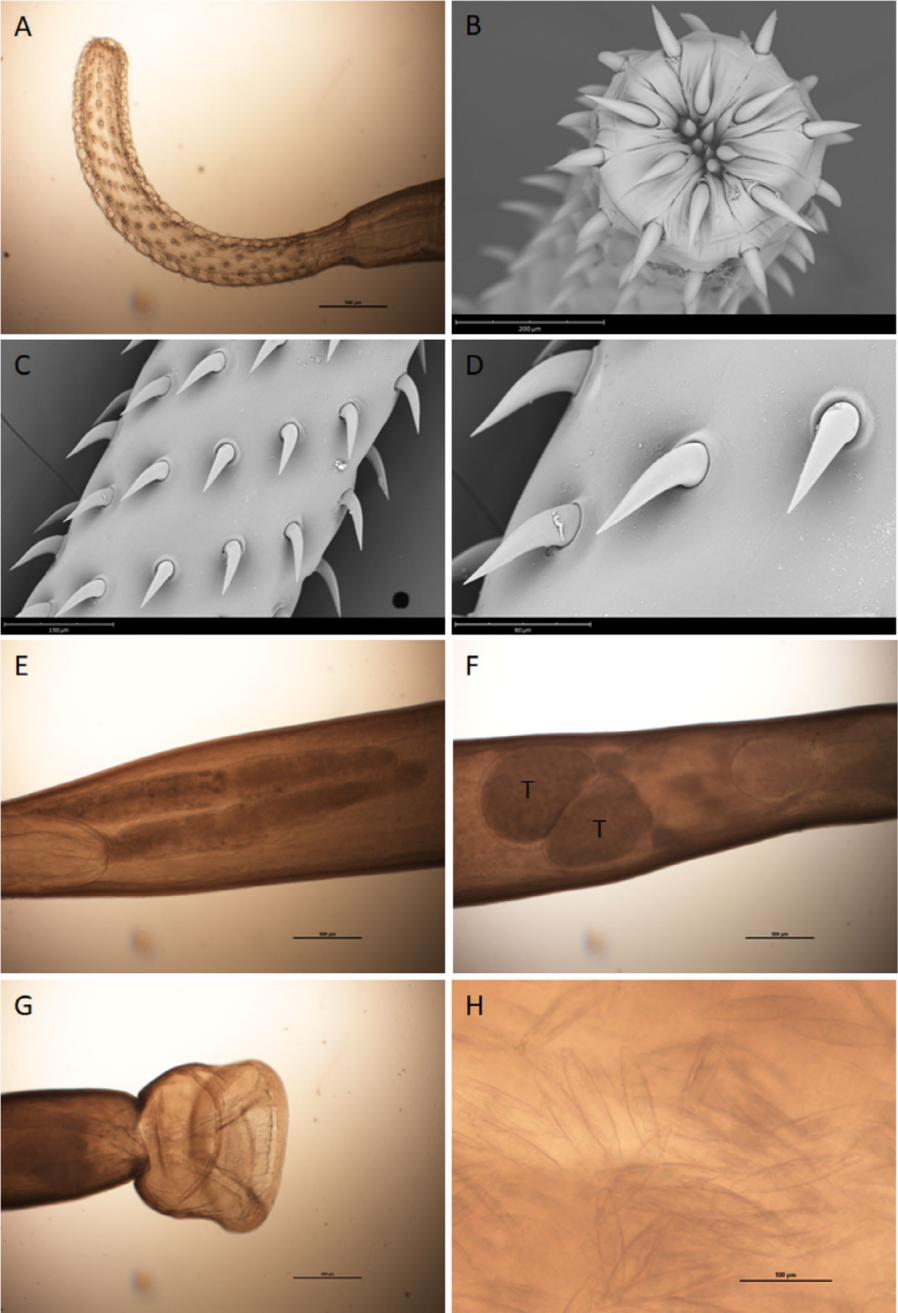
Light and scanning electron microscopy images of *Leptorhynchoides polycristatus* : (A) proboscis; (B) anterior hooks; (C) central hooks; (D) detail of hooks with striations and cuticular collar around the base (E) lemnisci; (F) mid‐body of male specimen, showing the appearance of testes (T); (G) everted bursa in male specimen; (H) detail of eggs in female specimen.

For scanning electron microscopy (SEM) analysis, formalin‐fixed specimens were washed three times in phosphate buffer, dehydrated through a graded ethanol series, subjected to critical point drying, sputter coated with gold palladium and observed using a Phenom XL G2 Desktop SEM (Thermo Fisher Scientific, Eindhoven, the Netherlands) operating at 5 kV. Morphological and morphometric characters as determined in light and scanning electron microscopy are consistent with the description of *Leptorhynchoides polycristatus* parasitizing sturgeons *Acipenser* spp. from the Caspian Sea (Amin et al. 2013). Observed morphological features were as follows: trunk cylindrical, slender, tapering towards both ends, rounder posteriorly. Proboscis long and slightly swollen anteriorly (Figure 3A), with 12–14 longitudinal rows of 19 hooks each. Apical hooks smaller and more curved (Figure 3B), central hooks longer (Figure 3C); basal hooks slightly decreasing in length and less curved. Hook surface with longitudinal striations and cuticular collar around base (Figure 3D). Lemnisci long, slender, unequal (Figure 3E). Female reproductive system simple; gonopore near terminal; eggs elongated, smooth, spindle shaped (Figure 3H). Table 1 summarises the morphometric measures of the analysed specimens.

The species *L. polycristatus* has never been reported in Italy, whereas another congeneric, *Leptorhynchoides plagicephalus*, was previously reported (Foata et al. 2004; Rossi et al. 1992) and related to reduced growth performances in heavily affected *A. naccarii* (Rossi et al. 1992). The two species differ in a number of morphological features: the number of hooks/row (19–20 vs. 22–24), the eggs width (markedly lower in *L. polycristatus* ), position of the longest hooks (anteriorly in *L. plagicephalus* , mid‐proboscis in *L. polycristatus* ), presence of a cuticular collar around the base of hooks and of a cuticular trunk collar (features absent in *L. plagicephalus* ) (Amin et al. 2013). In our specimens, the cuticular trunk collar was not observed; however, as pointed out in the original description of *L. polycristatus* , this feature may not be readily distinguishable or consistently observable (Amin et al. 2013).

## 6 Histology

**FIGURE 4:**
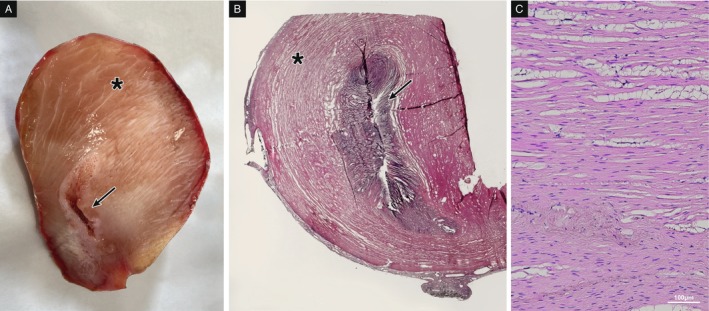
Histological examination of the stomach. (A) Macroscopical view of a sagittal slice of the stomach. Thickening of the muscular layer (asterisk) can be appreciated coupled with the reduction of the gastric lumen (arrow). (B) Histologically, the thickening of the muscle layer (asterisk) can be observed coupled with the reduction of the gastric lumen (arrow) (H&E, 0X). (C) Smooth muscle fibres appears well orientated and differentiated without cellular atypia (H&E, 10X), displacement of muscle fibres (clear spaces) is an artefact relatable to freezing.

At necropsy, samples of heart, spleen, ovary and stomach were collected and fixed in 10% neutral buffered formalin until further processing. Samples were then dehydrated through a graded ethanol–xylene series and embedded in paraffin. Sections of 3 μm were first deparaffinised, rehydrated and then stained with haematoxylin–eosin (H&E) for morphological examination. Spleen, heart and ovary showed extensive autolytic phenomena due to the freezing–thawing process. However, in the ovary, it was possible to identify primary and previtellogenic oocytes suggestive of an active gonad. Histological examination of the stomach revealed a proliferative lesion involving the stomach wall and constituted by smooth muscle fibres orientated in different planes and well differentiated. No cell atypia and limited mitotic activity was detected (Figure 4C). The proliferative mass developed eccentrically to the gastric lumen and was covered by serosa (Figure 4B). Based on the histological aspect, the proliferative lesion was consistent with leiomyoma.

## 7 Genetic Analysis

Genetic analyses were performed in order to try to assess the origin (wild or captive) of the specimen. A fin biopsy was performed in vivo when the specimen was moved to the aquaculture plant, and the DNA was extracted using the *DNeasy Blood & Tissue Kit* (Qiagen), following the ‘Quick‐Start Protocol’ for tissues. Subsequently, the DNA was stored at −20°C until further analysis.

The purity of the individual was detected by analysing the entire mitochondrial control region. This region was amplified, purified and sequenced as described in Congiu et al. (2011). Based on the comparison with the haplotypes already known for this species, each polymorphism was checked manually on the chromatogram using MEGAX (Kumar et al. 2018), and the Blast analysis matched with the *A. naccarii* haplotype 3. Further tests using species‐specific nuclear markers (Barmintseva and Mugue 2013; Boscari, Barmintseva, et al. 2014) confirmed the purity of the animal.

To determine the wild or captive origin of the specimen, a relatedness analysis was performed between the individual and the Italian captive breeding stock, which all Adriatic sturgeons employed in national restocking programmes descend from. Seventeen microsatellite *loci* were amplified following the conditions reported by Barca et al. (2022) and PCR products were genotyped at the BMR Genomics (external service, Padova, Italy, https://www.bmr‐genomics.it/). Allele scoring was performed using GENEMARKER software *v. 1.95* (SoftGenetics LLS) and the individual was successfully genotyped at 12 microsatellite *loci*.

**FIGURE 5:**
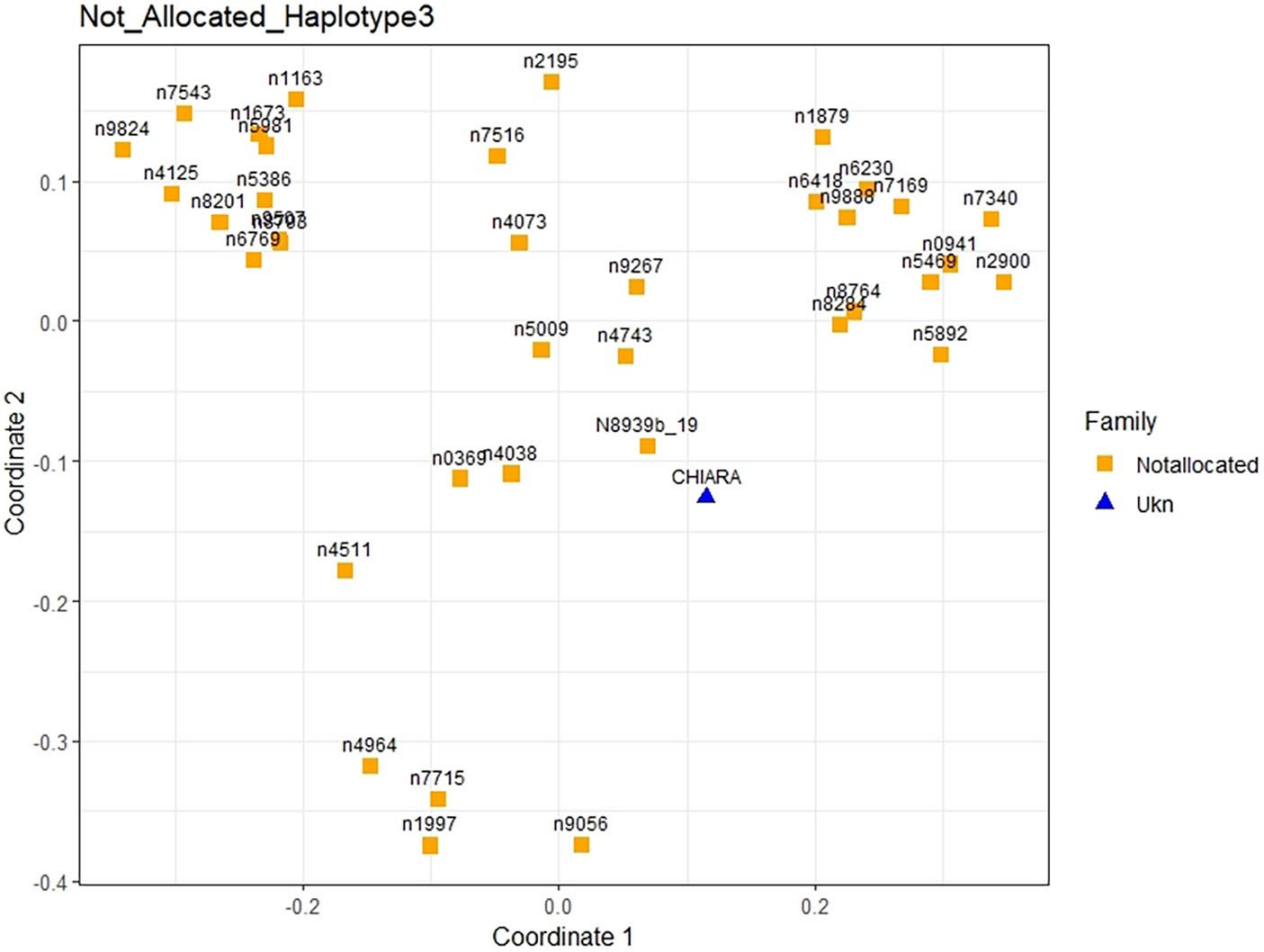
Results of multi‐dimensional scaling (MDS) analysis. The analysed subject (blue triangle labelled as ‘CHIARA’) did not cluster with any group of not allocated animals with the same haplotype.

The parental assignment was carried out using *BreedingSturgeons* software (Boscari, Pujolar, et al. 2014), specifically developed for tetraploid sturgeon species. Two different algorithms were applied: the standard band‐sharing measure and a weighted estimation of segregating alleles from different possible parent pairs and putative offspring. The specimen was not assigned to any parental pair, suggesting a wild origin. However, since some of the animals from the original reference stock are not present in our database, it is not possible to definitively rule out that the animal was generated in captivity using at least one unknown animal, though this is quite an unlikely occurrence. Therefore, to further explore the potential wild origin of the specimen under examination, a *multi‐dimensional scaling* (MDS) analysis was performed to visualise pairwise genetic distances among the individual and other unassigned captive animals with the same haplotype. The results (Figure 5) showed that the individual did not cluster with any group of not‐allocated animals. Additionally, the presence of a unique allele supports the likelihood of a wild origin.

## 8 Discussion

Infectious diseases are comprised among the threats to the sturgeon population recovery. Unfortunately, very few reports on the diverse pathogens affecting *Acipenser* spp. and *A. naccarii* specifically are available in the literature. This is because sturgeons are generally considered a robust fish species affected by few pathogens, especially during the long adult stage. This belief, coupled with an overall scarcity of specific diagnostic methods (namely molecular assay for viral diseases), makes disease reports in this species occasional (Mugetti et al. 2020). In this specific case, no viral pathogens were detected and no bacteriological analysis was possible due to long frozen storage. However, no gross lesions referable to bacterial diseases were observed. Laboratory analysis outcomes allowed the identification of the most probable cause of death that was linked to the almost complete obstruction of the gastric lumen that inevitably caused a feeding incapacity. Because of the mass's size and the emaciation status of the fish, a chronic process has been suspected. The massive infestation by *L. polycristatus* further exacerbated the nutritional deficiency. This represents the first description of an infestation with *L. polycristatus* in Italy, providing precious information on the Adriatic sturgeon parasitofauna.

Sturgeons are particularly vulnerable to neoplasia due to their longer lifespan, but very few cases are reported. Table 2 summarises available information from the literature. The first detection involved two immature *Acipenser transmontanus* specimens housed in a municipal aquarium. Both fish showed cutaneous lesions located at the level of the fins and at the ventral part of the body. Lesions were diagnosed as papilloma and were linked to mechanical stimulus due to the contact of the fish with the aquarium walls (Honma et al. 1999). Rezaie et al. (2018) described the histological features of a cardiac cavernous haemangioma observed in a diseased immature *Acipenser baerii*. The laboratory investigations were not able to explain the clinical signs that included skeletal malformation, severe emaciation, exophthalmoses and skin ulcers. However, the report represents the first detection of such neoplasia in Siberian sturgeon. Rahmati‐Holasoo et al. (2018) reported the clinical, imaging and histopathological features of nephroblastoma occurring in *Huso huso × Acipenser ruthenus* hybrids. Juvenile fish showed a monolateral or bilateral abdominal distension with a prevalence of 1%. In all fish examined at necropsy, coelomic cavities displayed the presence of large masses encompassing most of the visceral organs, including the swim bladder. Based on histological and immunohistochemical findings, masses were diagnosed as nephroblastoma. Despite not being able to identify the aetiology of the neoplasia, chemical carcinogenesis or genetic influences were suspected. Recently, cutaneous nerve sheath tumours were reported in farmed Russian sturgeon, *A. gueldenstaedtii* (Mandrioli et al. 2025). In this case, an immature adult showed several cutaneous masses at the level of the fins. As for previous reports, the development of the neoplasia was suspected to be related to genetic causes or to mechanical stimuli.

**TABLE 2:** Reported neoplasia in sturgeons.

Neoplasia	Fish species	Life stage	Setting	Reference
Papilloma	*Acipenser transmontanus*	Immature adult	Aquarium	Honma et al. (1999)
Haemangioma	*Acipenser baerii*	Immature adult – 3 years old	Aquaculture establishment	Rezaie et al. (2018)
Nephroblastoma	*Huso huso x Acipenser ruthenus*	Juveniles – 1 year old	Aquaculture establishment	Rahmati‐Holasoo et al. (2018)
Cutaneous nerve sheath tumour	*Acipenser gueldenstaedtii*	Immature adult	Aquaculture establishment	Mandrioli et al. (2025)

With reference to the mass in this study, it was identified histologically as being a leiomyoma, a neoplasia of the smooth muscle cells. Similar tumours have been reported in different organs in several fish species such as *Carassius auratus* (Çiltaş and Hisar 2005; Oryan et al. 2015; Uma et al. 2024), *Cyprinus carpio* (Vergneau‐Grosset et al. 2016), *Dicentrarchus labrax* (Iaria et al. 2020; Natale et al. 2020), *Micropterus salmoides* (Herman and Landolt 1975), *Mugil cephalus* (Singaravel et al. 2016), *Oncorhynchus tshawytscha* (Lumsden and Marshall 2003) and *Sardina pilchardus* (Ramos and Pelenteiro 2003). All these reports come from adult fish showing chronic disease or no disease depending on the affected organs. Notably, Iaria et al. (2020) described a gastric leiomyoma affecting a European sea bass broodstock. In that case, the neoplasia was detected during a routine health check and was not linked to disease yet. In our case, the mass caused occlusion of the stomach, impairing feed assumption and therefore the location rather than the neoplasia itself was the probable cause of the disease.

This case is the first report of neoplasia occurring in Adriatic sturgeon, *A. naccarii* , and can provide valuable information for future studies focused on diseases of this species. More reports should be encouraged in order to gather helpful information to acquire better knowledge on diseases affecting sturgeons. In conclusion, this report is a clear example of how investigating even a single wild fish specimen could provide important results on different ecological aspects.

## Author Contributions

**A. Marsella:** investigation, writing – original draft, conceptualization. **T. Pretto:** writing – review and editing, methodology, formal analysis, investigation. **C. Samassa:** writing – review and editing, methodology, formal analysis, writing – original draft, investigation. **P. Tedesco:** writing – review and editing, methodology, formal analysis, writing – original draft, investigation. **F. Pascoli:** writing – review and editing, investigation. **L. Congiu:** methodology, writing – review and editing, supervision, investigation. **A. Gustinelli:** methodology, writing – review and editing, supervision, investigation. **F. Borghesan:** investigation. **A. Toffan:** investigation, conceptualization, writing – review and editing, supervision, writing – original draft.

## Ethics Statement

No experimental procedures were performed on the fish. Handling and transportation of the examined fish were performed by trained professionals and in agreement with the current European and Italian regulations in force.

## Conflicts of Interest

The authors declare no conflicts of interest.

## Data Availability

Data used for the preparation of the present manuscript are herein reported. Any further information can be provided on reasonable request to Authors.
